# Genome-Wide RNAi Screening Identifies Novel Host Factors Involved in Influenza A Virus Infection in A549 Cells

**DOI:** 10.3390/v18030374

**Published:** 2026-03-17

**Authors:** Qingchao Zhang, Lifang Zhang, Xinmeng Yang, Wei Wang, Xiliang Wang, Chengyu Jiang, Fengming Huang, Yanli Zhang

**Affiliations:** 1 State Key Laboratory of Common Mechanism Research for Major Diseases, Department of Biochemistry and Molecular Biology, Institute of Basic Medical Sciences, Chinese Academy of Medical Sciences and Peking Union Medical College, Beijing 100005, China; 2 State Key Laboratory of Pathogens and Biosecurity, Beijing Institute of Microbiology and Epidemiology, Beijing 100071, China

**Keywords:** RNAi screening, influenza A virus, PR8, A549, drug repurposing

## Abstract

Influenza A virus (IAV) remains a major global health threat, and host-directed antivirals may help overcome rapid viral mutation and drug resistance. Here, we performed a genome-wide siRNA screen in A549 cells using cell viability as an integrated endpoint to identify host determinants of IAV (PR8/H1N1) infection. Using plate-normalized viability ratios, we identified 2134 genes with >40% viability change after infection (2048 UP and 86 DOWN; two-tailed *t*-test, *n* = 3; *p* < 0.05, FDR < 0.1). MetaCore pathway analysis showed enrichment of programs linked to host response and tissue injury control, including RAS-related signaling and multiple metabolic pathways such as estradiol, ubiquinone/mitochondrial redox, and benzo[a]pyrene/xenobiotic metabolism. DAVID Gene Ontology analysis further highlighted biological processes relevant to infection, including endocytosis, transcription, and translation, consistent with host pathways supporting viral replication. Benchmarking against meta-analyzed RNAi and CRISPR resources revealed that shared hits were enriched for translation, nucleocytoplasmic transport, and ER-Golgi trafficking, supporting external validity, whereas the large unique UP fraction was dominated by hormone metabolism, detoxification, and mitochondrial redox/CoQ pathways, consistent with viability-specific, tolerance-associated host response programs. Integrating the screen with DrugBank identified 174 druggable host genes corresponding to 345 candidate compounds. Together, these findings provide a systematic resource of host factors influencing H1N1 infection, improve understanding of influenza virus–host interactions, and offer a foundation for future development of host-directed antiviral strategies and drug repurposing efforts.

## 1. Introduction

Influenza poses a significant global health threat with limited effective therapeutic options. In humans, the influenza A virus (IAV) is responsible for most influenza infections, causing yearly seasonal influenza epidemics and, occasionally, also pandemics. Annual influenza epidemics lead to significant mortality, particularly among adults aged 65 years and older. According to the World Health Organization (WHO), influenza-associated deaths range from 290,000 to 600,000 annually [1]. Influenza viruses are negative-sense, single-stranded, multisegmented RNA viruses belonging to the Orthomyxoviridae family. They exhibit a high tendency for random mutations (antigenic drift) and specific gene segment reassortment (antigenic shift), which continually generate new influenza strains. Currently used drugs to combat influenza are targeted to viral proteins, such as amantadine (an M2 ion channel blocker) and oseltamivir (a neuraminidase inhibitor) [2,3,4]. While this approach is undoubtedly the most logical and direct, the high mutation rates exhibited by RNA viruses frequently lead to the swift emergence of resistant viral strains [5]. We must acknowledge the potential danger of influenza variants gaining the ability to sustain transmission among humans, possibly leading to new global pandemics. There is an urgent need for innovative intervention strategies to effectively combat potential influenza pandemics in the future.

The IAV exploits the host’s cellular machinery for its replication and propagation, making host factors potential therapeutic targets. Compared to targeting viral proteins, selectively targeting host factors that are temporarily dispensable for the host but essential for the IAV life cycle offers an alternative approach to delay or even prevent the development of drug resistance. An example of this host-directed approach is DAS181, a recombinant sialidase, which has been assessed in various clinical trials [6,7]. By inhibiting these contributing host factors, we can effectively hinder the viral lifecycle, reduce virus production, and ultimately mitigate virus-induced cell death and pathogenesis.

To identify potential antiviral drug targets, it is crucial to fully understand which host factors are involved during virus infection. RNA interference (RNAi) is a reliable method for identifying essential genes in diseases [8,9]. Genome-wide RNAi screenings typically use virus replication as the measure of interference and have been utilized to identify host genes involved in the replication of the IAV [10,11,12,13,14,15,16]. IAV infection of lung epithelial cells leads to cell death through mechanisms such as apoptosis, necroptosis, and pyroptosis, which contribute to inflammation, IAV lethality, barrier loss, and pathogenesis [17,18,19,20]. Therefore, we used cell death as the primary indicator to develop a genome-wide RNAi screening technique to identify host factors involved in IAV infection and repurposable drugs against IAV. Through this method, we discovered host factors involved in the infection of PR8 virus in A549 lung epithelial cells.

Additionally, data mining of public databases like DrugBank has become increasingly common for identifying candidate drugs that might interact with validated target genes [21,22]. By combining RNAi screening with searches in DrugBank, we developed an effective method for identifying repurposable drugs.

In this study, we employed genome-wide RNAi screening and identified 2134 host genes that affected cell viability after PR8 virus infection by over 40%. Clustering and pathway analysis using MetaCore and DAVID provided insights into the functional roles of these genes. We then analyzed these genes using the DrugBank database, identifying 345 approved or clinically investigated drugs targeting 174 genes.

## 2. Materials and Methods

### 2.1. Cell Culture and Virus Infection

The A549 human lung adenocarcinoma cell line was sourced from the Cell Culture Center at Peking Union Medical College and cultured in Ham’s F12 medium (Hyclone, Logan, UT, USA) supplemented with 10% fetal bovine serum (FBS, Gibco, Grand Island, NY, USA). The H1N1 influenza virus A/Puerto Rico/8/34 (PR8) was preserved in Beijing Institute of Microbiology and Epidemiology, and propagated by inoculation into specific-pathogen-free embryonated fowl eggs via the allantoic route. All experiments involving live virus were performed in biosafety level 2 facilities. A549 cells were transfected with siRNAs targeting 18,602 coding genes, followed by infection with PR8 24 h post-transfection. Cell viability was assessed 48 h post-infection using the MTS assay (catalog no. G3582, Promega, Madison, WI, USA).

### 2.2. Genome-Wide siRNA Screen

A genome-wide siRNA library targeting 18,602 human genes (three siRNAs targeting each gene in a mix), along with negative-control (NC) siRNAs, was acquired from RiboBio (Guangzhou, China). A549 cells were plated into 96-well plates and transfected with siRNA (100 nM) using Lipofectamine RNAiMAX reagent (Invitrogen, Carlsbad, CA, USA). After 24 h of transfection, the A549 cells were infected with PR8 (MOI 3.0) or treated with an equal volume of allantoic fluid (AF). Cell viability was measured 48 h post-infection using the MTS assay. Cell viability values were normalized by calculating the ratio with respect to the viability of cells transfected with NC siRNA in the same plates as follows:

$$normalized\;cell\;viability=\frac{{siRNA}^{PR8}/{siRNA}^{AF}}{{NC}^{PR8}/{NC}^{AF}}$$

where siRNA^PR8^ represents the PR8-infected siRNA group, siRNA^AF^ represents the AF-treated siRNA group, NC^PR8^ represents the PR8-infected NC group, and NC^AF^ represents the AF-treated NC group. This method corrects for baseline impacts on cell viability by comparing infected to mock-infected conditions and normalizing the data relative to NC siRNA. Such normalization effectively differentiates virus-specific factors from general viability effects, thus substantially reducing the possibility of erroneously identifying common housekeeping or cell cycle-related genes as false positives.

For statistical testing, each group was measured in *n* = 3 replicate wells, and *p*-values were calculated using a two-tailed *t*-test by comparing the cell viability values of the siRNA group against the corresponding NC group on the same plates. To correct for multiple hypothesis testing across genes, false discovery rates (FDRs) were computed by adjusting *p*-values using the Benjamini–Hochberg method. A two-tailed *p*-value < 0.05 with FDR < 0.1 was considered statistically significant. Genes associated with normalized cell viabilities that increased or decreased by more than 40% were selected for further analysis. Detailed information about the host genes identified in the genome-wide RNAi screen is provided in Table S1 in the Supplemental Material.

### 2.3. Gene Enrichment and Drugs Repurposing

The top hits from the siRNA screen were functionally annotated and categorized using pathway and Gene Ontology (GO) enrichment analyses in MetaCore web-based platform(Clarivate Analytics, https://portal.genego.com/; accessed on 19 August 2024) and the DAVID platform [23,24]. For drug repurposing, candidate compounds targeting the identified genes were retrieved from the DrugBank database [21,22]. Candidate drugs were ranked by the number of screened genes they targeted, with drugs targeting the largest number of genes ranked highest. In cases where multiple drugs targeted an equal number of genes, we further prioritized based on *p*-values, with smaller *p*-values indicating higher significance and thus higher priority rankings.

## 3. Results

### 3.1. Genome-Wide RNAi Screening of Host Genes

**Figure 1:**
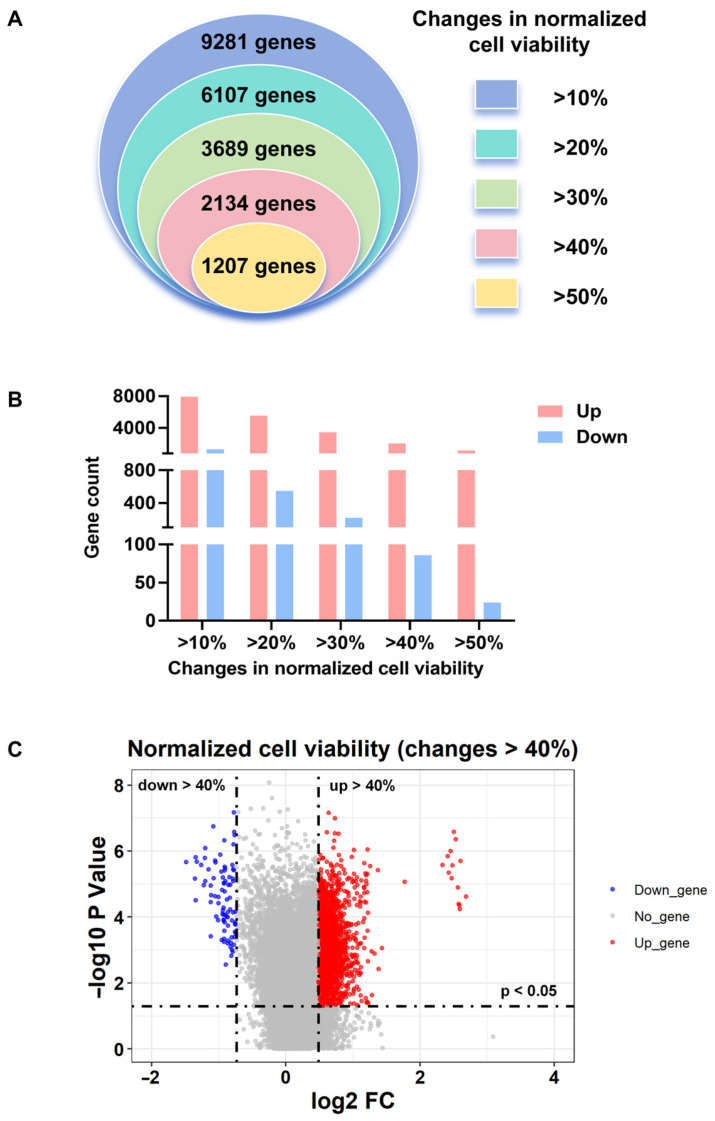
Genome-wide RNAi screening to identify essential host factors for PR8 infection. Counts of genes associated with (**A**) changes, and (**B**) increase or decrease in cell viability of more than 10%, 20%, 30%, 40%, or 50% in the genome-wide RNAi screens. (**C**) The volcano diagrams of genes targeted by RNAi treatments that altered cell viability by more than 40% following PR8 infection. (The horizontal axis represents the log2 fold change in normalized cell viability for various gene knockdowns. The vertical axis indicates the statistical significance of changes in cell viability. Each point on the plot corresponds to a specific gene. Gray dots denote siRNA-targeted genes that either altered cell viability by less than 40% or were not statistically significant following PR8 infection. Red dots represent UP genes whose knockdown increased cell viability, while blue dots indicate DOWN genes whose knockdown decreased cell viability.

**Figure A1:**
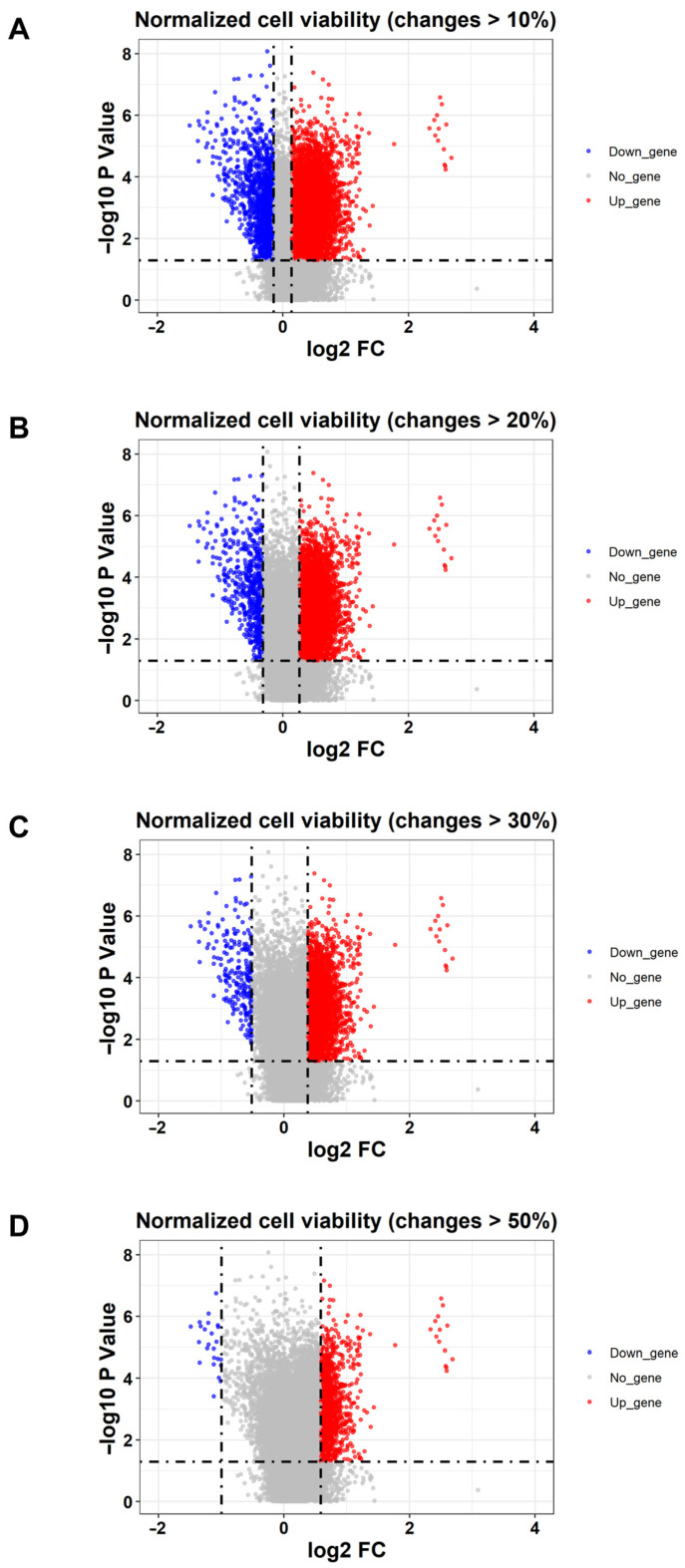
Genome-wide RNAi screening to identify host factors influencing PR8 infection. Volcano plots depict the results of genome-wide RNAi screens in A549 cells infected with PR8 influenza virus. Cell viability was measured following gene knockdown by siRNA. Panels show results for different cutoff thresholds of cell viability change: (**A**) >10%, (**B**) >20%, (**C**) >30%, and (**D**) >50%. Plot Details: X-axis (log2 FC): Log2-fold change in normalized cell viability relative to control (siRNA targeting a non-targeting control). Positive values indicate increased cell viability upon gene knockdown; negative values indicate decreased cell viability. Y-axis (−log10 *p*-value): Negative logarithm (base 10) of the *p*-value from statistical testing of cell viability changes. Higher values indicate greater statistical significance. The horizontal dashed line indicates the significance threshold. Data points: Each point represents a gene targeted by a specific siRNA. Gray dots: Genes where knockdown resulted in cell viability changes below the indicated threshold ((**A**): 10%, (**B**): 20%, (**C**): 30%, (**D**): 50%) or changes that were not statistically significant. Red dots: Genes whose knockdown significantly increased cell viability, suggesting these genes may promote virus infection. Blue dots: Genes whose knockdown significantly decreased cell viability, suggesting these genes may restrict virus infection or be essential for cell survival.

A total of 18,602 human encoding genes were screened using RNAi, and 9281 host genes were identified that affect cell viability by more than 10% following PR8 infection (Figure 1A,B, *p* < 0.05, FDR < 0.1). Among these 9281 significant genes, RNAi treatments altered cell viability with PR8 infection by more than 20% for 6107 genes, 30% for 3689 genes, 40% for 2134 genes, and 50% for 1207 genes (Figure 1A). The histogram (Figure 1B) illustrates the number of genes targeted by RNAi treatments that either increased (UP genes, red) or decreased (DOWN genes, blue) cell viability by more than 10%, 20%, 30%, 40%, and 50%, with specific counts being 7949, 5557, 3470, 2048, and 1183 for UP genes and 1332, 550, 219, 86, and 24 for DOWN genes, respectively. There are substantially more UP genes—ranging from 6 to 50 times as many—whose knockdown increases cell viability compared to DOWN genes whose knockdown decreases cell viability. This difference is especially pronounced in the group showing most significant changes in cell viability (Figure 1B). Genes targeted by RNAi treatments that altered cell viability by more than 40% (Figure 1C) and by more than 10%, 20%, 30%, and 50% (Figure A1) following PR8 infection are presented as volcano plots.

### 3.2. Functional Clustering and Pathway Analysis

**Figure 2:**
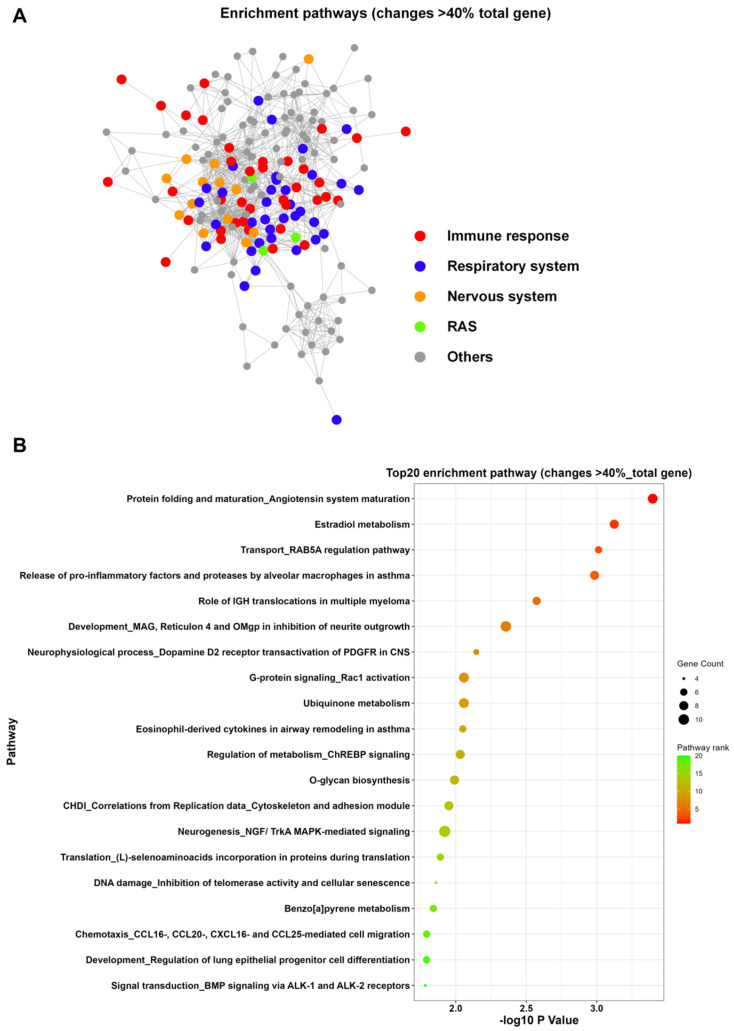
Functional enrichment analysis of pathways for essential host genes associated with PR8 infection. (**A**) Enriched pathways associated with 2134 genes, as analyzed using Metacore. Enrichment was visualized using the Enrichment Map application in Cytoscape (version3.4.0). Pathways are presented as nodes, while the node color indicates the significance of pathways and node size reflects the object count enriched in the pathways. The connection between pathways is based on shared objects. (**B**) Functional enrichment of the top 20 significant pathways for 2134 genes. The size and color of the nodes indicate the gene count and pathway rank, respectively.

**Figure A2:**
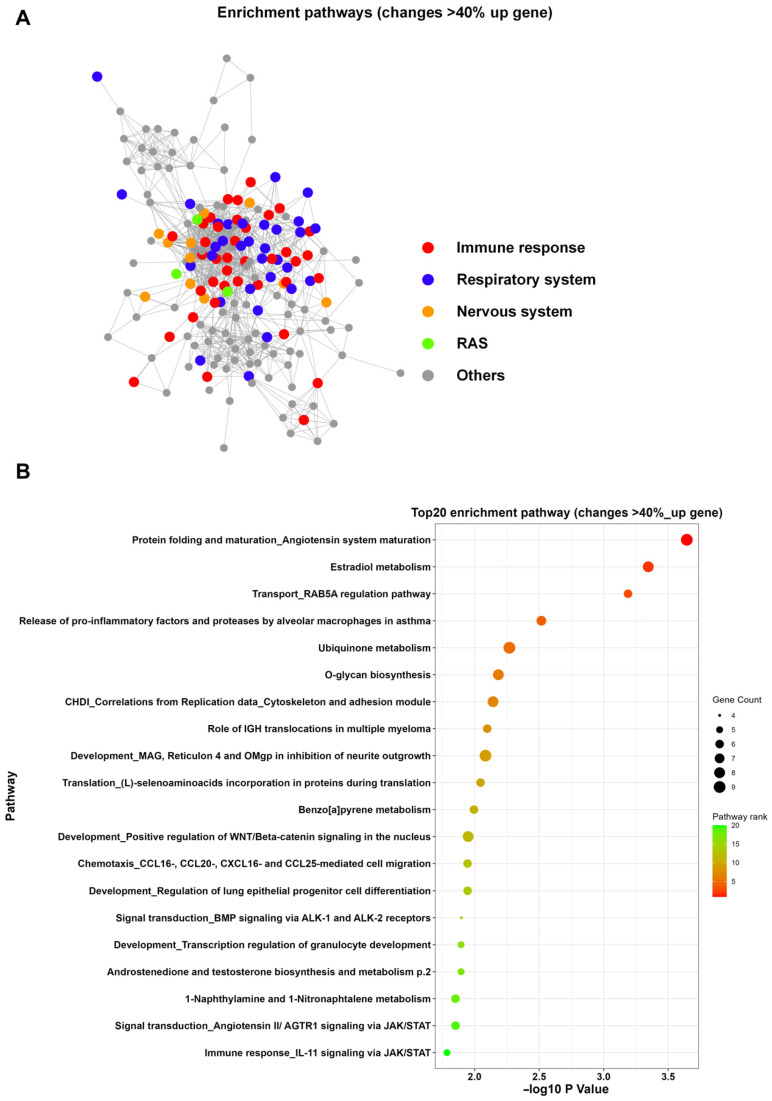
Functional enrichment analysis of host cell pathways modulated by genes whose knockdown increased cell viability by more than 40% following PR8 infection. (**A**) Network representation of enriched pathways. Functional enrichment analysis was performed using Metacore on the 2048 UP genes (genes whose knockdown increased cell viability by >40%). The resulting enriched pathways were visualized as a network using the Enrichment Map plugin in Cytoscape. Nodes: Represent individual enriched pathways. Node Color: Indicates the statistical significance of the pathway enrichment (from red to gray, more significant to less significant). Node Size: Reflects the number of genes from the input list (2048 UP genes) that are annotated to that pathway (gene count). Edges (Connections): Connect pathways that share a significant number of genes, indicating functional relatedness. (**B**) Top 20 most significantly enriched pathways. The top 20 enriched pathways from the Metacore analysis of the 2048 UP genes are shown, ranked by statistical significance (false discovery rate < 0.05). Y-axis: Pathway name. X-axis (−log10 *p*-value): Negative logarithm (base 10) of the *p*-value, indicating the statistical significance of pathway enrichment. Higher −log10 *p*-values represent greater significance. Dot Size: Represents the number of genes from the input list (2048 UP genes) associated with the pathway (gene count). Larger dots indicate more genes. Dot Color: Represents the rank of the pathway based on significance (from red to green, top rank to lower rank).

**Figure A3:**
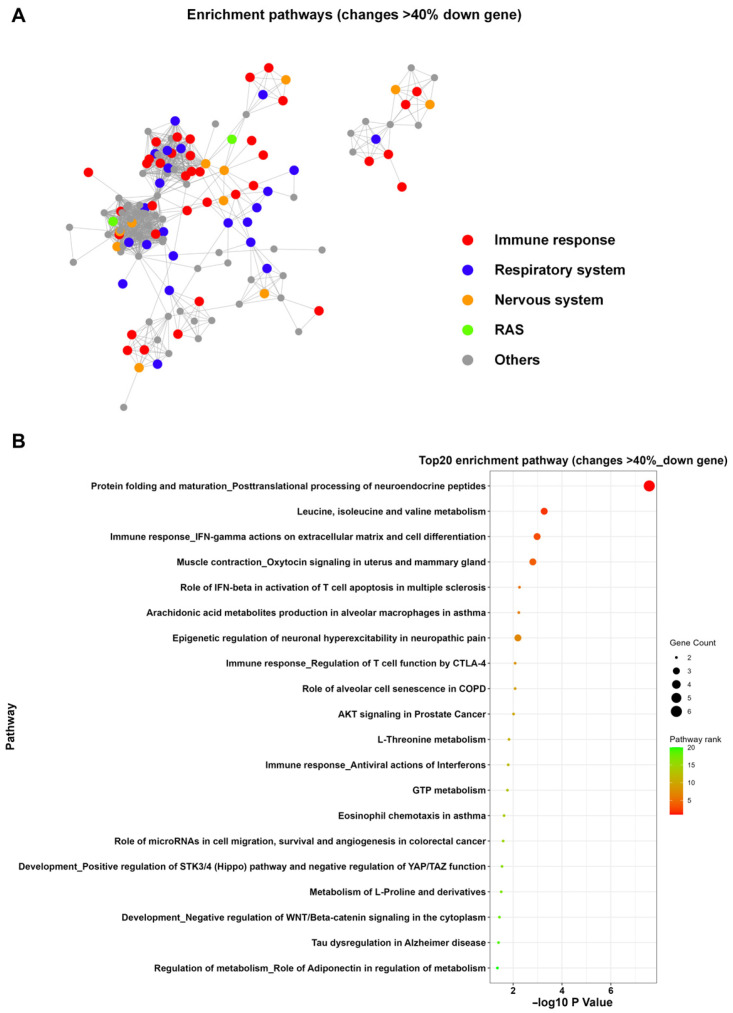
Functional enrichment analysis of host cell pathways modulated by genes whose knockdown decreased cell viability by more than 40% following PR8 infection. (**A**) Network representation of enriched pathways. Functional enrichment analysis was performed using Metacore on the 86 DOWN genes (genes whose knockdown decreased cell viability by >40%). The resulting enriched pathways were visualized as a network using the Enrichment Map plugin in Cytoscape. Nodes: Represent individual enriched pathways. Node Color: Indicates the statistical significance of the pathway enrichment (from red to gray, more significant to less significant). Node Size: Reflects the number of genes from the input list (86 DOWN genes) that are annotated to that pathway (gene count). Edges (Connections): Connect pathways that share a significant number of genes, indicating functional relatedness. (**B**) Top 20 most significantly enriched pathways. The top 20 enriched pathways from the Metacore analysis of the 86 DOWN genes are shown, ranked by statistical significance (false discovery rate < 0.05). Y-axis: Pathway name. X-axis (−log10 *p*-value): Negative logarithm (base 10) of the *p*-value, indicating the statistical significance of pathway enrichment. Higher −log10 *p*-values represent greater significance. Dot Size: Represents the number of genes from the input list (86 DOWN genes) associated with the pathway (gene count). Larger dots indicate more genes. Dot Color: Represents the rank of the pathway based on significance (from red to green, top rank to lower rank).

**Figure A4:**
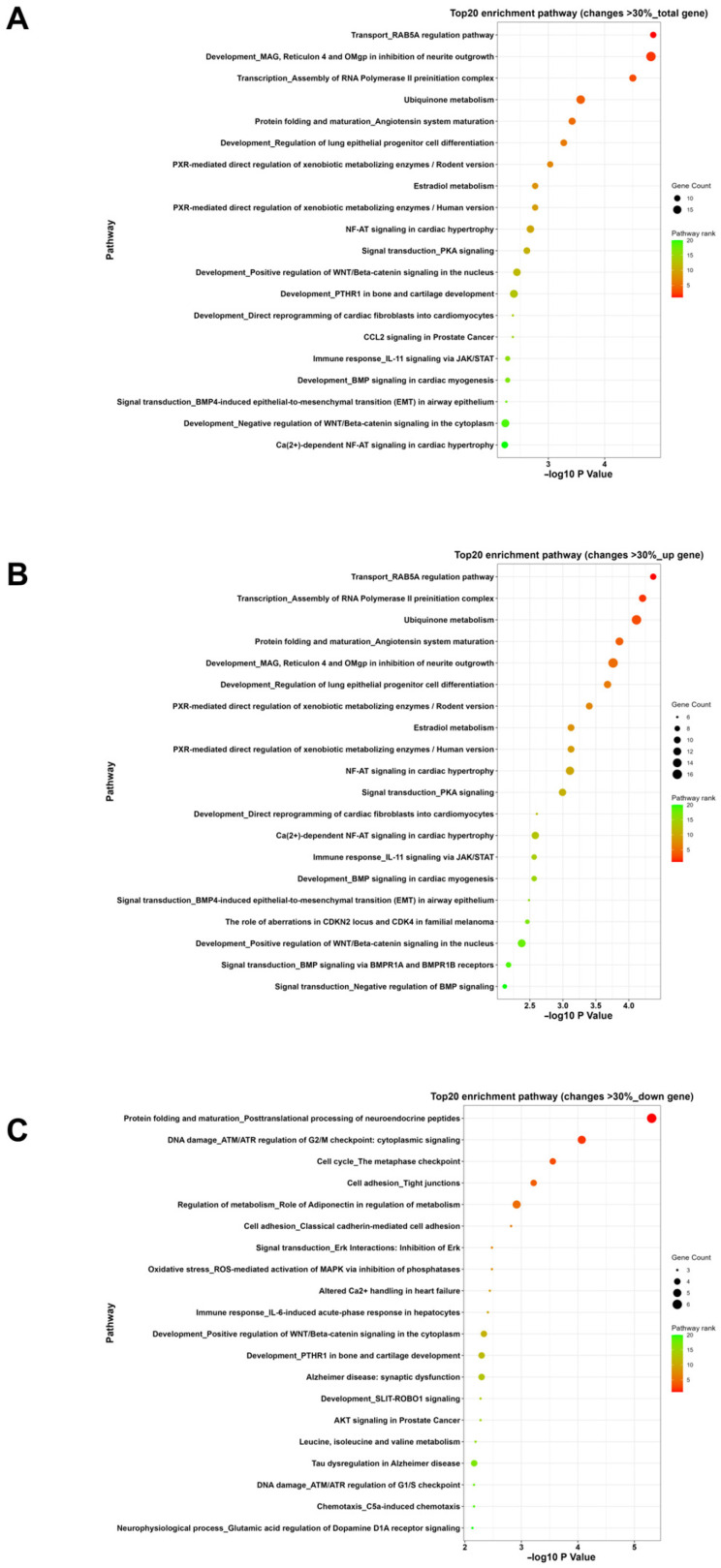
Functional enrichment analysis of pathways for essential host genes whose knockdown changed cell viability by more than 30% during PR8 infection. Functional enrichment of the top 20 significant pathways for the (**A**) 3689 genes whose knockdown changed cell viability by more than 30%, (**B**) 3470 UP genes whose knockdown increased cell viability by more than 30%, (**C**) 219 DOWN genes whose knockdown decreased cell viability by more than 30%, as analyzed using Metacore. (false discovery rate < 0.05). The size and color of the nodes indicate the gene count and pathway rank, respectively.

Analysis using the Metacore database indicated that the 2134 genes were mostly enriched in pathways related to the immune response, respiratory system, nervous system, and renin-angiotensin system (RAS) (Figure 2A, Table S2). Among the top 20 functional enrichment pathways, three were associated with the metabolism of estradiol (E2), ubiquinone, and benzo[a]pyrene (BaP), four with protein translation, maturation, transport, and translocation, five with signal transduction, and two with the immune response (Figure 2B, Table S3). Specifically, the pathways related to metabolism and protein translation, maturation, transport, and translocation were predominantly enriched with the UP genes whose knockdown increased cell viability. In contrast, the immune response pathways were mainly enriched with the DOWN genes whose knockdown decreased cell viability (Figure A2 and Figure A3, Tables S4 and S5). We also analyzed the 3689 genes that altered cell viability by more than 30% in the RNAi screen following PR8 infection using the Metacore database (Figure A4, Table S6). The transcription pathway related to the assembly of the RNA polymerase II preinitiation complex was also enriched among the UP genes, along with pathways involved in protein translation, maturation, transport, and translocation. This indicates that viruses extensively commandeer the host’s gene expression machinery for successful replication (Figure A4B, Table S7).

**Figure 3:**
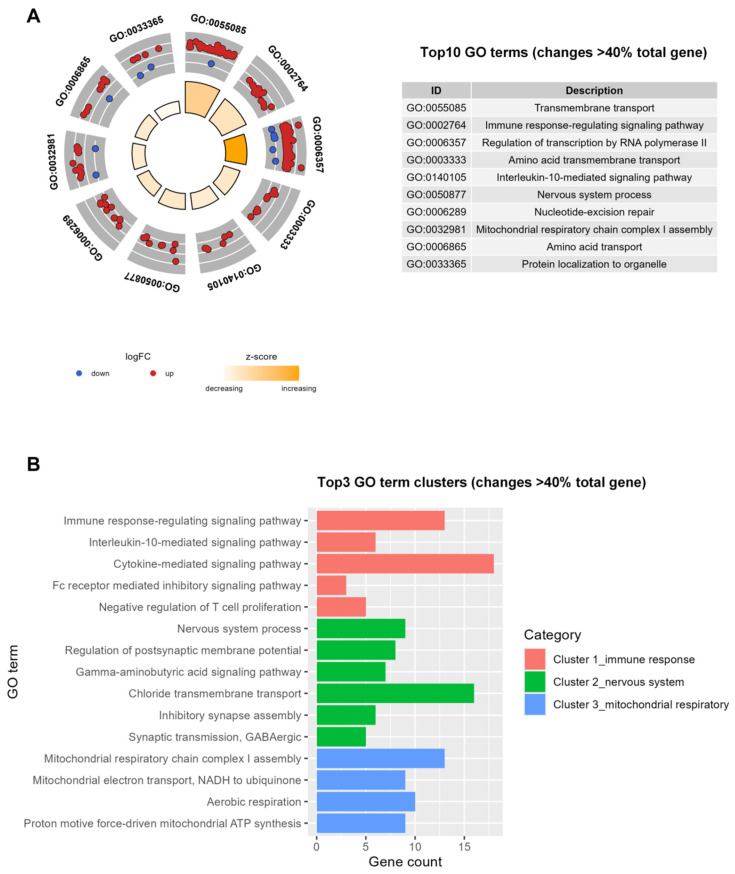
Gene ontology of the essential host genes associated with PR8 infection analyzed by DAVID and the GOplot package. (**A**) GOCircle plot showing the number of genes in each GO term. The inside rings are a bar plot, with the height representing the significance of the term (−log10 adjusted *p*-value). The colors of the inside rings represent the Z-score. The outside rings show the change levels (logFC) for each gene in the GO term. Each dot in the outside rings represents one gene from the GO term, red dots show UP genes whose knockdown increases cell viability and blue dots show DOWN genes whose knockdown decreases cell viability. (**B**) Top 3 GO term clusters for the 2134 genes.

The DAVID database analysis revealed that the Top 10 most significantly enriched Gene Ontology (GO) terms for Biological Processes (BP) for the 2134 genes included transmembrane transport, immune response-regulating signaling pathway, regulation of transcription by RNA polymerase II, amino acid transmembrane transport, interleukin-10-mediated signaling pathway, nervous system process, nucleotide-excision repair, mitochondrial respiratory chain complex I assembly, amino acid transport, and protein localization to organelle (Figure 3A, Table S8). The Top 3 GO term clusters for the 2134 genes were related to immune response, nervous system processes, and mitochondrial respiratory functions (Figure 3B, Table S9).

### 3.3. Cross-Screen Comparison Highlights Shared Replication Dependencies and Viability-Specific Host Responses

To benchmark our viability-based RNAi screen against prior influenza host-factor studies that primarily relied on reporter signals, viral protein abundance, or other replication-centric readouts, we compared our stringent hits (2048 UP and 86 DOWN genes; >40% change in normalized viability during PR8/H1N1 infection) with two meta-analysis resources integrating genome-wide RNAi and CRISPR screens. The RNAi-focused meta-analysis curated 153 high-evidence proviral genes (and only two antiviral genes supported by multiple RNAi screens), whereas the CRISPR-focused meta-analysis by information content (MAIC) study generated ranked lists of host dependency and host restriction factors [25,26]. Because our assay quantifies the integrated survival consequence of infection, we expected partial concordance with replication-centered screens together with additional outcome-modifying pathways preferentially detected by a viability endpoint.

**Figure 4:**
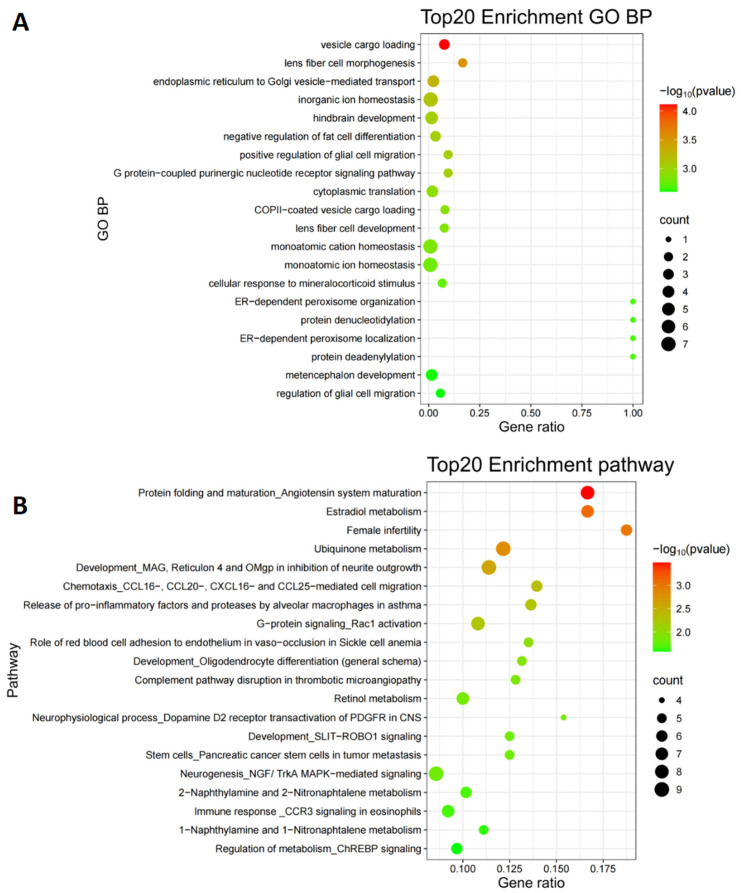
Functional enrichment Functional enrichment profiles distinguish conserved overlap hits from viability-enriched unique hits. (**A**) Top 20 enriched Gene Ontology (GO) Biological Process (BP) terms for the 61 overlap genes shared with published host-factor resources. (**B**) Top 20 enriched MetaCore pathway maps for the 1987 unique UP genes identified only by our viability-based screen. In both panels, the size and color of the nodes indicate the gene count and the significance of pathways, respectively.

Consistent with this expectation, 61 UP genes overlapped with published proviral resources (9/153 from the RNAi meta-analysis and 52/500 from the top MAIC host-dependency factors supported by >4 screens) (Table S10). These shared candidates were enriched in conserved influenza-relevant host programs, including translation and endoplasmic reticulum (ER)-Golgi secretory-pathway trafficking, indicating that our viability endpoint recovers a reproducible subset of canonical host dependency machinery (Figure 4A, Table S11). In contrast, the overlap with published host restriction factors was minimal: among the 86 DOWN genes, only PABPC3 (Poly(A)-binding protein cytoplasmic 3), which is involved in mRNA stability and translation regulation, overlapped with the top MAIC host-restriction list (448 genes supported by >2 screens), highlighting a smaller contribution of host restriction programs in our viability-based screen.

Importantly, concordance was far from exhaustive. 1987 of the 2048 UP genes were unique to our screen after excluding the 61 shared genes, indicating that a survival-based readout captures additional host determinants that are less frequently recovered by assays optimized for replication-centric endpoints (Table S10). MetaCore pathway-map enrichment of these 1987 unique UP genes revealed hormone-metabolic pathways (e.g., angiotensin system maturation, E2 metabolism), xenobiotic metabolism (e.g., BaP-related pathways), mitochondrial redox/ubiquinone metabolism, and multiple stress/inflammatory signaling and migration modules (e.g., Rac1, macrophage pro-inflammatory factor/protease release, and CCL/CXCL-mediated chemotaxis) (Figure 4B; Table S12). Together, these results support the expected pattern for a viability-based assay—retaining a measurable signature of conserved replication dependencies while additionally capturing infection-outcome determinants linked to stress handling, metabolic–redox adaptation, and host-response remodeling.

### 3.4. Selection of Candidate Drugs

**Figure 5:**
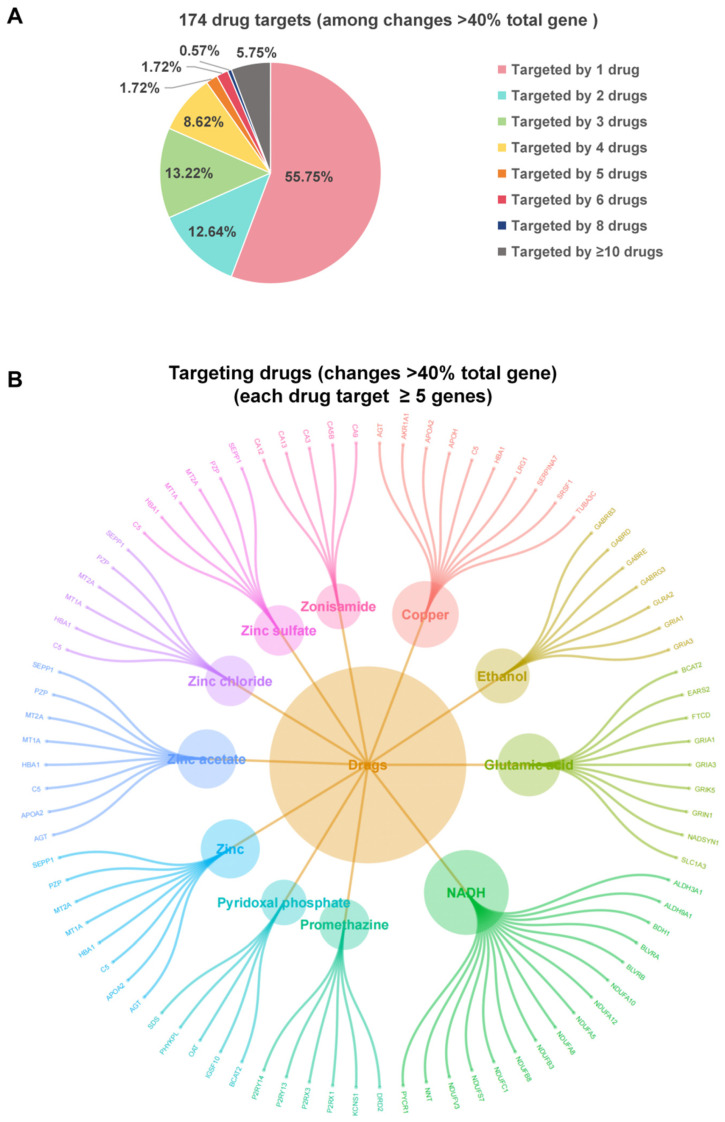
Selection of candidate drugs using the DrugBank database. Among the 2134 host genes, 174 genes can be targeted by drugs in the DrugBank Database. (**A**) The pie-chart illustrates the proportion of genes that can simultaneously be targeted by 1, 2, 3, 4, 5, 6, 8, and ≥10 drugs, with each sector representing the respective percentage. (**B**) 11 drugs can simultaneously target ≥5 genes among the 174 genes.

**Figure A5:**
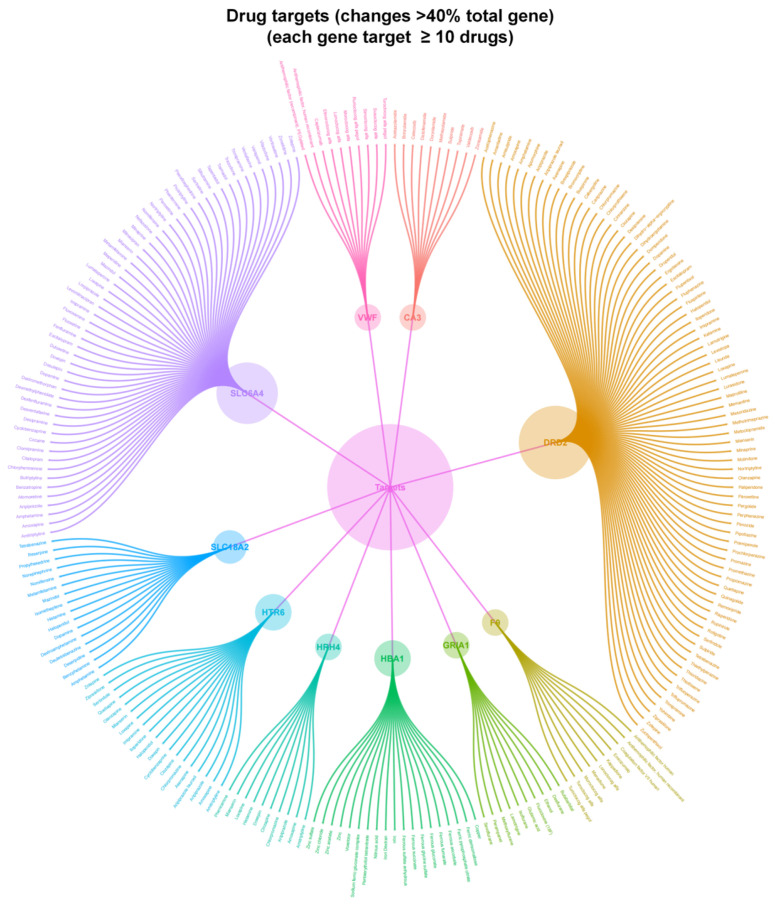
Network representation of host genes targeted by multiple drug candidates. This figure illustrates the relationship between host genes identified in the RNAi screen and potential drug repurposing candidates. Outer Circles: Represent the ten host genes whose activity can be modulated by ten or more drug candidates. These genes were identified as potential therapeutic targets based on the RNAi screen results (genes altering cell viability by >40%). Connections (Lines): Connect each of the ten host genes to the drug candidates predicted to target them. This network highlights potential drug repurposing opportunities for influenza treatment by targeting key host factors.

Among the 2134 host genes that altered cell viability by more than 40% in the RNAi screen following H1N1 virus infection, we identified 174 genes targeted by a total of 345 drug candidates using the DrugBank database. Following the strategy detailed in the Materials and Methods section, these 345 drug candidates were prioritized, and a corresponding rank list was generated (Table S13). Of these 174 drug targets, 97 (55.75%) are targeted by a single drug, 22 (12.64%) by two drugs, 23 (13.22%) by three drugs, 15 (8.62%) by four drugs, 3 (1.72%) by five drugs, 3 (1.72%) by six drugs, 1 (0.57%) by eight drugs, and 10 (5.75%) by ten or more drugs (Figure 5A). The ten drug targets that can be simultaneously targeted by ten or more drugs are shown in Figure A5. Among these, SLCGA4 and DRD2 can be targeted by 56 and 79 drugs, respectively. Additionally, among the 345 drug candidates, 11 can simultaneously target five or more genes. Four zinc-based medications used to treat zinc deficiency are among the 11 drugs identified. These include zinc, zinc acetate, zinc chloride, and zinc sulfate (Figure 5B).

## 4. Discussion

Viruses hijack host metabolic pathways to enable replication, relying on specific cellular proteins that become potential therapeutic targets. While traditional antiviral strategies focus on viral proteins, targeting host factors provides an alternative approach against influenza, particularly given the rapid development of drug resistance.

### 4.1. Comparison with Previous RNAi and CRISPR Screens

In this study, we established a whole-genome RNAi platform to identify druggable host genes involved in H1N1 infection using A549 lung epithelial cells—a well-characterized and cost-effective model for large-scale influenza research [13,16]. Multiple independent RNAi and CRISPR screens have investigated host determinants of influenza replication, typically using viral or reporter protein expression as the readout [10,11,12,13,14,25,26,27,28]. Visualization of infected cells with viral protein-specific antibodies or the use of recombinant viruses expressing fluorescent or enzymatic reporters (e.g., GFP, luciferase) allows quantification of infection. The choice of readout and post-infection timing defines the lifecycle stage examined: early protein detection reflects viral entry and translation, whereas late protein detection encompasses genome replication and assembly. The selected readout and the timing of the post-infection assay determine which aspects of the viral lifecycle are examined. For instance, assessing a viral protein expressed early in the infection process tracks the virus through entry, uncoating, and protein expression stages. Conversely, detecting viral proteins that require prior replication of the viral genome (‘late’ genes) includes the replication phase in the examined viral life-cycle steps.

However, when a virus infects a host, ‘viral replication hijacking’ and the ‘host restriction’ to reduce replication are not the only processes occurring within the cell. The host can also limit the damage caused by the viral burden through a response known as ‘disease tolerance’ without necessarily changing viral load. All these cellular activities can be reflected in cell viability in vitro. Therefore, we designed our RNAi screening platform to use cell death as the readout, as shown in our previous study [27]. This approach enables us to evaluate the efficacy of siRNA by observing the overall cellular response and outcomes to PR8 virus infection, encompassing all aspects of ‘virus replication hijacking’, ‘host restriction’, and ‘disease tolerance’, rather than solely focusing on viral replication.

### 4.2. Mechanistic Classification of Viability-Based RNAi Hits

In our viability-based influenza RNAi screen, we classified hits into three mechanistic classes that apply to both UP and DOWN genes. Class I (host dependency genes) comprises conserved host machinery repeatedly exploited by influenza viruses for replication and implicated across influenza host-factor screens (e.g., RNA processing/gene-expression machinery, translation and ribosome components, nucleocytoplasmic transport, and ER–Golgi/COPII trafficking). Perturbation of Class I genes is therefore expected to reduce productive infection and typically manifests as UP genes (increased viability upon knockdown). Class II (host restriction genes) includes antiviral immune pathways, such as interferon-associated defenses. Disruption of these determinants generally exacerbates infection-associated damage and thus typically yields DOWN genes (decreased viability upon knockdown). Class III (tolerance-associated host response genes) encompasses outcome-modifying programs—including metabolic and redox resilience, hormone/xenobiotic detoxification, and stress/signaling axes—that shift the balance between cellular injury and tolerance during infection. Accordingly, Class III genes can appear as either UP genes (when knockdown attenuates cytopathic injury amplification) or DOWN genes (when knockdown compromises stress adaptation and tolerance capacity).

### 4.3. IAV Life Cycle-Related Class I Host Dependency Programs Enriched Among UP Genes

The IAV life cycle comprises entry, nucleocytoplasmic trafficking, nuclear transcription/replication, cytosolic translation, and ER–Golgi secretory trafficking for viral membrane protein maturation and virion assembly. Consistent with these requirements, our UP-gene set contains, at both the gene and enrichment levels, multiple Class I host dependency modules that map to these core steps.

#### 4.3.1. IAV Entry Related Endosomal Trafficking as Class I Programs

RAB guanosine triphosphatases are key regulators of endosomal trafficking and maturation, with RAB5 and RAB7 controlling early and late endosomes, respectively—both essential for viral entry [29]. Notably, RAB5A appears in the external high-confidence intersection between the RNAi-153 proviral list and the CRISPR top-500 host-dependency list (24-gene intersection), supporting RAB-mediated trafficking as a reproducible influenza-relevant dependency layer across screening modalities. Although RAB5A itself was not recovered as a gene-level hit under our stringent overlap criteria, our pathway-level analysis independently prioritized a RAB5-centered module; ‘Transport_RAB5A regulation’ ranked among the top enriched pathways (rank 3) and implicated multiple associated components including EGF, RABGEF1, and ANKRD27 (Varp). Collectively, perturbation of this RAB5 regulatory axis is expected to impair endosomal trafficking and maturation required for productive entry, consistent with a conserved Class I (host dependency) program captured by our viability endpoint.

#### 4.3.2. Nucleocytoplasmic Transport and RNA Processing as Class I Programs

The overlap UP-gene set also contained key determinants of nucleocytoplasmic transport and RNA processing, exemplified by RAN and the spliceosome-associated factor SF3B2. These functions align closely with core influenza biology: IAV requires efficient nuclear import of vRNPs and polymerase components, nuclear export of viral mRNAs and assembled vRNPs, and extensive engagement of host RNA-processing machinery to support productive infection. The recurrence of nuclear transport and RNA-processing factors within the overlap subset therefore reinforces that our viability-based screen captures a reproducible signature of Class I host dependency programs that are consistently implicated across independent influenza host-factor screens.

#### 4.3.3. Viral Protein Translation and ER–Golgi/COPII Secretory Trafficking as Class I Programs

Consistent with prior influenza RNAi and CRISPR screens in which protein translation/translation initiation emerged among the most overrepresented functional categories [25,26,30], GO Biological Process enrichment analysis of our 61 overlap UP genes was dominated by cytosolic translation and ER–Golgi/COPII secretory-pathway trafficking, two conserved host-dependency modules required for productive infection. In our overlap set, representative translation-associated genes include EIF3H and ribosomal components RPL30 and RPL34, which collectively sustain host translational capacity and are plausibly co-opted to support viral protein synthesis. This translation signature therefore aligns with Class I (host dependency genes).

A second prominent overlap signature was early secretory trafficking, reflected by enrichment of ER to Golgi vesicle-mediated transport, vesicle cargo loading, and COPII-coated vesicle cargo loading. These processes are mechanistically linked to influenza biology because the viral membrane proteins HA, NA, and M2 are synthesized on ER-bound ribosomes, inserted into the ER membrane, and must traverse the ER-Golgi network for maturation and delivery to the plasma membrane. In our overlap set, genes consistent with this trafficking theme include CTAGE9 and TBC1D20, which are associated with ER/secretory pathway organization and vesicle dynamics, as well as SCAP, a key ER-to-Golgi cargo adaptor in the COPII-mediated transport of the SREBP complex. Notably, TBC1D20 encodes an ER-associated RAB GTPase-activating protein (RabGAP) that can modulate RAB activity states and thereby influence ER exit-site function and downstream trafficking. Together, the recurrence of translation and ER-Golgi/COPII terms in the overlap subset supports a conserved influenza-relevant dependency layer captured by our viability endpoint.

### 4.4. Unique UP Genes Are Enriched for Class III Tolerance-Associated Host Response Programs

Pathway-map enrichment of the 1987 unique UP genes was dominated by Class III (tolerance-associated host response programs). The top maps clustered into hormone–metabolic and endocrine themes (e.g., angiotensin system maturation and estradiol metabolism), xenobiotic detoxification programs (e.g., BaP metabolism and naphthylamine/nitronaphthalene-related pathways), and mitochondrial redox/CoQ biology (e.g., ubiquinone metabolism). These Class III themes were accompanied by host-response modules associated with inflammatory injury, including pathways related to macrophage-driven release of pro-inflammatory factors/proteases and CCL/CXCL-mediated chemotaxis. Below, we further examine several representative pathways highlighted by this enrichment analysis.

#### 4.4.1. Angiotensin System Maturation as a Class III Pathway

MetaCore enrichment highlighted an ‘Angiotensin system maturation’ theme within the unique UP pathways, driven by the mapping of AGT (angiotensinogen) to angiotensin peptide network objects in the pathway map (e.g., angiotensin I/II and angiotensin-(1–7)). This signal is most consistent with a Class III mechanism because the RAS is an endocrine/paracrine cascade that influences infection outcome primarily by modulating vascular-barrier function, inflammatory injury, and redox stress, rather than serving as a discrete host dependency for a specific step of viral replication.

Within this system, AGT is the upstream precursor from which multiple bioactive angiotensin peptides are generated through stepwise proteolytic processing. A key regulatory checkpoint is the opposing roles of ACE and ACE2: ACE generates angiotensin II (Ang II), a potent effector peptide that signals mainly through AT1R/AT2R to promote vasoconstriction, increased vascular permeability, oxidative stress, and pro-inflammatory/pro-fibrotic responses, whereas ACE2 cleaves Ang II to angiotensin-(1–7), shifting signaling toward the protective Mas receptor axis and thereby restraining RAS activation by reducing Ang II levels [31]. Notably, our group previously demonstrated in a lethal avian influenza model that H5N1 infection rapidly downregulates pulmonary ACE2, is accompanied by elevated systemic Ang II, and that ACE2 deficiency accelerates mortality and exacerbates lung injury due to RAS imbalance in infected mice [32]. Importantly, because AGT feeds the entire angiotensin cascade, the emergence of an ‘angiotensin maturation’ signal in our viability-based screen likely reflects host programs that tune the ACE/ACE2–Ang II/Ang-(1–7) equilibrium and ultimately influence survival under influenza challenge, even when direct effects on viral gene expression are not the primary determinant.

#### 4.4.2. E2 Metabolism as a Class III Pathway

The enriched E2 metabolism pathway included multiple cytochrome P450 (CYP) enzymes and UDP-glucuronosyltransferases (UGTs), specifically CYP1A2, CYP1B1, CYP3A4, CYP2D6, UGT2A1, UGT1A10, UGT2B11, and UGT1A4.

E2, the most potent estrogen, is produced by the ovaries and predominates before menopause. It is metabolically inactivated and excreted via urine or feces. In estrogen metabolism, E2 is first hydroxylated by CYP enzymes (e.g., CYP1A2/CYP3A4-mediated 2-hydroxylation and CYP1B1-mediated 4-hydroxylation) and subsequently conjugated by UGT enzymes of the 1A, 2A, and 2B families to promote clearance via urine or feces. Prior studies have shown that E2 acts as a potent anti-inflammatory hormone and attenuate influenza pathogenesis by modulating pulmonary inflammatory responses and immune cell recruitment without directly reducing viral replication [33,34,35]. In our screen, knockdown of the above CYP/UGT genes increased viability by >40%, consistent with the possibility that reduced metabolic inactivation/clearance may increase effective E2 signaling and thereby shift the injury–tolerance balance toward improved survival, in line with a Class III mechanism.

#### 4.4.3. BaP Metabolism Pathway as a Class III Pathway

The enrichment of the BaP metabolism pathway provides another example of Class III among unique UP genes. This pathway included both phase I CYP enzymes and phase II conjugation enzymes, specifically CYP1A2, CYP1B1, CYP3A4, UGT1A10, UGT1A9, and UGT1A6. BaP is a representative polycyclic aromatic hydrocarbon (PAH) that is biologically inert per se but can be bioactivated by CYP enzymes (e.g., CYP1A1) to generate reactive metabolites [36]. The lung is a major exposure site for environmental and tobacco-smoke PAHs, and BaP exposure has been reported to exacerbate the severity of viral respiratory illness. Moreover, BaP metabolites were shown to markedly suppress influenza virus-induced interferon responses in mammalian cells [37]. In our RNAi screen, knockdown of the phase I enzymes CYP1A2, CYP1B1, and CYP3A4 increased viability by >40%, consistent with the possibility that reducing BaP bioactivation may attenuate metabolite-associated cellular stress and immune perturbation during infection. Notably, knockdown of the phase II enzymes UGT1A10, UGT1A9, and UGT1A6 also increased viability by >40%. As UGTs mediate detoxifying conjugation of BaP-derived reactive metabolites [38], this directional phenotype suggests that the net effect of perturbing different steps of the BaP metabolic network on infection outcome may be context-dependent in our viability endpoint, reinforcing BaP metabolism as an outcome-modifying Class III pathway.

#### 4.4.4. Ubiquinone/Complex I Pathway as a Class III Outcome-Modifying Program

The enrichment of the ubiquinone metabolism pathway further supports the predominance of Class III among unique UP genes, implicating mitochondrial redox and stress handling as key determinants of infection outcome. This pathway enriched multiple mitochondrial NADH dehydrogenase (Complex I) subunits, specifically NDUFA8, NDUFB3, DAP13, NDUFS7, NDUFC1, NDUFV3, NDUFA5, NDUFA10, and NDUFB8. Mammalian Complex I is a large multi-subunit enzyme composed of 45 subunits [39,40] and catalyzes electron transfer from NADH to ubiquinone (coenzyme Q, CoQ10), thereby initiating oxidative phosphorylation and shaping mitochondrial redox homeostasis. CoQ10 is not only an essential component of the respiratory chain but also serves antioxidant functions, and its depletion has been associated with dysregulated redox balance in acute and chronic illness settings [41]. In our RNAi screen, knockdown of the above Complex I subunit genes increased normalized viability by >40% during PR8 infection. Given that influenza infection can strongly remodel host metabolism and mitochondrial function, a plausible interpretation is that reducing complex I-dependent mitochondrial electron flux to ubiquinone can mitigate infection-associated mitochondrial stress (including dysregulated reactive oxygen species signaling and downstream cell death pathways) and thereby improve cell survival. This phenotype reflects regulation of the injury–tolerance balance captured by a viability endpoint and is most consistent with the Class III mechanism it.

As an experimental validation example, one representative hit from our screen, CRBN (cereblon), has been independently validated in vivo. We showed that lung-specific Crbn knockout(Sftpc-Cre; Crbnflox/flox) significantly attenuates IAV-induced acute lung injury in mice, consistent with a protective effect mediated by the CRBN–AMPK–MAPK axis [42]. Mechanistically, because CRBN can promote proteasomal degradation of AMPKα, Crbn deficiency is expected to increase AMPK abundance and constitutively activate AMPK signaling, which may in turn suppress downstream MAPK activation and mitigate excessive inflammatory responses during IAV infection. Importantly, this CRBN–AMPK–MAPK pathway exemplifies a Class III mechanism: modulating host stress and inflammatory signaling shifts the injury–tolerance balance and improves survival outcomes, rather than reflecting a core host dependency required for a discrete step of viral replication.

### 4.5. Role of Nervous System and Drug Target Insights

We further investigated the 2134 host genes that altered cell viability by more than 40% in the RNAi screen as potential drug targets using the DrugBank database (www.DrugBank.ca/). This analysis identified 345 drug candidates targeting 174 of these genes. Among them, ten genes were targeted by ten or more compounds; notably, two nervous system-related genes, SLC6A4 and DRD2, were targeted by 56 and 79 drugs, respectively—mainly antidepressants and antipsychotics.

Current antidepressants primarily enhance monoamine neurotransmission by inhibiting the presynaptic transporters that reuptake neurotransmitters released from presynaptic terminals. SLC6A4, a sodium- and chloride-dependent high-affinity serotonin transporter (also known as 5-hydroxytryptamine transporter, 5-HTT, or SERT), is located in presynaptic neuronal membranes and mediates serotonin (5-HT) clearance from the synaptic cleft. Increased synaptic serotonin concentrations are believed to underlie the antidepressant effect. Several drug classes target SLC6A4, including the widely prescribed selective serotonin reuptake inhibitors (SSRI) such as fluoxetine, sertraline, citalopram, and fluvoxamine, as well as serotonin–norepinephrine reuptake inhibitors (SNRI) like venlafaxine, duloxetine, and desvenlafaxine. Other antidepressants acting on SLC6A4 include serotonin modulators (trazodone, vortioxetine, vilazodone) and tricyclic or tetracyclic compounds (e.g., clomipramine, imipramine, nortriptyline, amitriptyline, and mianserin) (Figure A5).

Most older antipsychotics act as DRD2 antagonists. First-generation drugs such as chlorpromazine, haloperidol, and fluphenazine, and second-generation agents including clozapine, risperidone, and olanzapine, primarily block D2 receptors. In contrast, third-generation antipsychotics—aripiprazole, brexpiprazole, and cariprazine—introduced in the 2000s, function as partial dopamine receptor agonists, providing more balanced dopaminergic modulation (Figure A5).

Our findings indicate that the nervous system plays a crucial role in PR8 infection, as demonstrated by the enrichment of neural-related pathways in the Metacore and DAVID analyses. Consistently, many of the identified drugs are nervous system agents. Previous studies have reported neurological complications following IAV infection, including headaches, depression, seizures, and, in severe cases, encephalopathy and Reye’s syndrome. Although the underlying mechanisms remain unclear, they may involve viral invasion of the CNS, immune activation, cytokine storms, or other indirect effects [43,44,45]. Our results suggest an additional mechanism: SLC6A4 and DRD2, key neuronal transporter and receptor genes, were found to beUP genes whose knockdown enhanced cell viability. This implies that their products may be hijacked by the virus to support replication, thereby disrupting normal neuronal functions and contributing to neurological manifestations. Targeting such virus-facilitating host genes could potentially influence both influenza pathogenesis and its neurological sequelae, warranting further investigation.

## 5. Conclusions

Overall, our study highlights the potential of genome-wide RNAi screening to uncover novel host factors and therapeutic targets for influenza virus infection. The findings provide a valuable resource for understanding host factors involved in influenza pathogenesis and developing targeted therapies.

## Data Availability

No new data were created.

## Supplementary Materials

The following supporting information can be downloaded at: https://www.mdpi.com/article/10.3390/v18030374/s1; Table S1: Host genes identified in the genomewide RNAi screen; Table S2: Mostly enriched pathway categories (changes >40% total genes); Table S3: Top20 enriched pathways (changes >40% total genes); Table S4: Top20 enriched pathways (changes >40% up genes); Table S5: Top20 enriched pathways (changes >40% down genes); Table S6: Top20 enriched pathways (changes >30% total genes); Table S7: Top20 enriched pathways (changes >30% up genes); Table S8: Top10 enriched GO Biological Process (BP) terms (changes >40% total genes); Table S9: Top3 enriched GO term clusters (changes >40% total genes); Table S10: Benchmarking of viability-based UP hits against published proviral host-factor resources; Table S11: Top 20 enriched GO Biological Process (BP) terms for shared proviral UP genes (overlap, *n* = 61); Table S12: Top 20 enriched MetaCore pathway maps for unique UP genes (*n* = 1,987); Table S13: 345 drugs and its 174 drug targets (changes >40% total genes).

## Abbreviations

The following abbreviations are used in this manuscript:

IAV	Influenza A virus
RNAi	RNA interference
PR8	A/Puerto Rico/8/34
WHO	World Health Organization
NC	negative control
AF	allantoic fluid
GO	Gene Ontology
BP	Biological Processes
E2	estradiol
BaP	benzo[a]pyrene
CYP	cytochrome P450
UGT	UDP-glucuronosyltransferases
PAH	polycyclic aromatic hydrocarbon
CoQ10	Coenzyme Q
5-HTT	5-hydroxytryptamine transporter
5-HT	serotonin
SSRI	serotonin reuptake inhibitors
SNRI	serotonin–norepinephrine reuptake inhibitors

## Appendix A

This figure displays the top 20 most significantly enriched pathways (false discovery rate < 0.05) identified by Metacore analysis for three gene sets. (A) All genes with >30% viability change (3689 genes): Functional enrichment analysis of the combined set of genes whose knockdown resulted in either increased or decreased cell viability by more than 30%. (B) UP genes (>30% increased viability, 3470 genes): Functional enrichment analysis of genes whose knockdown increased cell viability by more than 30%, suggesting these genes may promote virus infection. (C) DOWN genes (>30% decreased viability, 219 genes): Functional enrichment analysis of genes whose knockdown decreased cell viability by more than 30%, suggesting these genes may restrict virus infection or be essential for cell survival. Plot Details (Applicable to all panels A, B, and C): Y-axis: Pathway name. X-axis (−log10 *p*-value): Negative logarithm (base 10) of the *p*-value, indicating the statistical significance of pathway enrichment. Higher −log10 *p*-values represent greater significance. Dot Size: Represents the number of genes within the analyzed gene set (3689 in A, 3470 in B, or 219 in C) that are associated with the pathway (gene count). Larger dots/bars indicate more genes. Dot Color: Represents the rank of the pathway based on significance (from red to green, top rank to lower rank).
